# The drug interaction potential of daprodustat when coadministered with pioglitazone, rosuvastatin, or trimethoprim in healthy subjects

**DOI:** 10.1002/prp2.327

**Published:** 2018-03-09

**Authors:** Stephen Caltabiano, Kelly M. Mahar, Karyn Lister, David Tenero, Ramiya Ravindranath, Borut Cizman, Alexander R. Cobitz

**Affiliations:** ^1^ Metabolic Pathways GlaxoSmithKline Collegeville Pennsylvania; ^2^ Clinical Pharmacology Modeling & Simulation GlaxoSmithKline Upper Merion Pennsylvania; ^3^ Clinical Pharmacology Science & Study Operations GlaxoSmithKline Collegeville Pennsylvania; ^4^ Clinical Statistics GlaxoSmithKline Bangalore India

**Keywords:** Chronic kidney disease, drug interaction, phase I

## Abstract

This study was conducted to evaluate the likelihood of daprodustat to act as a perpetrator in drug–drug interactions (DDI) with the CYP2C8 enzyme and OATP1B1 transporter using the probe substrates pioglitazone and rosuvastatin as potential victims, respectively. Additionally, this study assessed the effect of a weak CYP2C8 inhibitor, trimethoprim, as a perpetrator of a DDI with daprodustat.

This was a two‐part study: Part A assessed the effect of coadministration of daprodustat on the pharmacokinetics of pioglitazone and rosuvastatin in 20 subjects; Part B assessed the coadministration of trimethoprim on the pharmacokinetics of daprodustat in 20 subjects.

Coadministration of 100 mg of daprodustat with pioglitazone or rosuvastatin had no effect on the plasma exposures of either probe substrate. When trimethoprim was coadministered with 25‐mg daprodustat plasma daprodustat AUC and *C*_max_ increased by 48% and 28%, respectively. Additionally, AUC and *C*_max_ for the metabolite GSK2531401 were decreased by 32% and 40%, respectively. *C*_max_ for the other metabolites was slightly decreased (~8–15%) but no changes in AUC were observed.

As 100‐mg daprodustat exceeds the planned top therapeutic dose, interaction potential of daprodustat as a perpetrator with substrates of the CYP2C8 enzyme and OATP1B1 transporters is very low. Conversely, daprodustat exposure (AUC and *C*_max_) is likely to increase moderately with coadministration of weak CYP2C8 inhibitors.

**Abbreviation**
AEs: adverse events
BMI: body mass index
CKD: chronic kidney disease
CYP: cytochrome
DDI: drug–drug interactions
DMPK: drug metabolism and pharmacokinetics
EPO: erythropoietin
ESA: erythropoiesis‐stimulating agent
GCP: Good Clinical Practice
HDD: hemodialysis dependent
HIF: hypoxia inducible factor
NDD: nondialysis dependent
PHI: prolyl hydroxylase inhibitor
PK: pharmacokinetic
SAE: serious adverse events

## Introduction

Daprodustat (GSK1278863) is an orally active, small molecule hypoxia‐inducible factor (HIF) prolyl hydroxylase inhibitor (PHI) that is currently in Phase 3 clinical studies. HIF‐PHIs are an emerging new class of agents under investigation for the treatment of anemia associated with chronic kidney disease (CKD) and work by stimulating erythropoiesis through inhibition of HIF‐prolyl hydroxylase domain enzymes (PHD1, PHD2, PHD3). This activity results in the accumulation of HIF*α* transcription factors leading to increased transcription of HIF‐responsive genes, stimulating components of the natural response to hypoxia. During hypoxia, the PHD enzymes are inhibited, resulting in the accumulation of unhydroxylated HIF*α* subunits, which dimerize with HIF*β* subunits to effect the transcription of HIF‐responsive genes, including erythropoietin (EPO) and others involved in increasing oxygen availability and utilization. Other functions regulated by HIFs include iron metabolism and utilization, angiogenesis, extracellular matrix metabolism, apoptosis, energy and glucose metabolism, vascular tone, cell adhesion, and motility (Haase 2013; Schmid and Jelkman 2016).

In two separate 4‐week clinical studies, daprodustat has demonstrated dose‐dependent increases in hemoglobin levels in hemodialysis‐dependent (HDD) and non–dialysis‐dependent (NDD) patients with anemia associated with CKD (Holdstock et al. 2016a). In NDD patients who were naïve to erythropoietin‐stimulating agent (ESA) treatment, an oral dose of 5 mg once daily led to a mean increase in hemoglobin of 1 gm/dL over 4 weeks. In HDD subjects switched from ESA treatment, daprodustat, at 5 mg once daily, maintained mean hemoglobin levels over the 4‐week treatment period. These data suggest that daprodustat may be an alternative to currently available ESAs for treatment of anemia associated with CKD. These results have been confirmed in two 24‐week clinical studies (Cobitz et al. 2016; Holdstock et al. 2016b).

The cytochrome (CYP) P450 enzymes that are involved in the oxidative metabolism of daprodustat have been evaluated both in vitro (human liver microsomes) and in clinical studies (Johnson et al. 2013). Results from these studies suggest that CYP2C8 is the primary enzyme involved with CYP‐mediated metabolism of daprodustat. In addition, in in vitro studies using human liver microsomes, daprodustat inhibited CYP2C8 with an IC_50_ value of 21 *μ*mol/L, while no inhibition was observed with other CYPs up to 100 *μ*mol/L. An initial clinical drug–drug interaction (DDI) study with the potent inhibitor gemfibrozil resulted in 4‐ and 19‐fold increases in daprodustat *C*_max_ and AUC, respectively (Johnson et al. 2013). In in vitro transporter assays, daprodustat inhibited OATP1B1‐mediated transport with an IC_50_ value of 6 *μ*mol/L. Therefore, in order to provide guidance on the coadministration of daprodustat with drugs that are substrates of CYP2C8 or OATP1B1, this study was conducted to assess the effect of daprodustat as a perpetrator of a DDI at doses up to 100 mg, on the pharmacokinetics of probe substrates for CYP2C8 and OATP1B1 (i.e., pioglitazone and rosuvastatin, respectively). Furthermore, as daprodustat is metabolized by CYP2C8, the effect of a weak CYP2C8 inhibitor (i.e., trimethoprim) on the pharmacokinetics of daprodustat was assessed.

## Materials and Methods

### Study design and endpoints

This study was designed as a two‐part study to assess the potential for daprodustat to affect the pharmacokinetics of rosuvastatin and pioglitazone, and the potential for trimethoprim to affect the pharmacokinetics of daprodustat (Study NCT02371603 on ClinicalTrials.gov registry):

1. Part A was an open‐label, randomized, single oral dose, two‐way cross‐over design that assessed the effects of a single, oral dose of 100 mg daprodustat on the pharmacokinetics of a single, oral dose of 15 mg pioglitazone and a single, oral dose of 10 mg rosuvastatin administered as a cocktail in healthy male and female subjects;
2. Part B was an open‐label, single sequence, two‐way cross‐over design to assess the effect of steady‐state trimethoprim (200 mg administered orally twice daily for 3 days) on the pharmacokinetics of a single, oral dose of 25 mg daprodustat in healthy, adult male and female subjects.

The study was conducted in accordance with Good Clinical Practice (GCP) guidelines and the 2008 Declaration of Helsinki, and was approved by an independent ethics committee (Midlands Independent Review Board, 8417 Sante Fe Drive, Suite 100, Overland Park, Kansas, 66212). Written informed consent was obtained from each subject before enrolment. The study was conducted at a single Clinical Unit (Quintiles Phase One Services LLC, 6700 West 115^th^ Street, Overland Park, Kansas, 66211).

### Participants

After the initial screening (up to 4 weeks prior to randomization), eligible male and female subjects were enrolled into the study. The inclusion criteria considered subjects declared healthy by a responsible and experienced physician based on medical history, physical examination, laboratory tests, and cardiac monitoring, with a body weight ≥ 50 kg and a body mass index (BMI) of 19–29.9 kg/m^2^. Subject eligibility criteria included females of non–child‐bearing potential or of child‐bearing potential confirmed to be using one of the required contraceptive methods.

Exclusion criteria included evidence of a significant abnormality on 12‐lead ECG at screening (including QTc interval > 450 msec), use of prescription or nonprescription drugs within 7 days (or 14 days if the drug was a potential enzyme inducer) or 5 half‐lives (*t*"), whichever was longer; history of regular use of tobacco‐ or nicotine‐containing products; peptic ulcer disease; preexisting conditions interfering with normal gastrointestinal anatomy or motility; history of drug allergy; recent participation in a clinical trial or administration of an investigational product; or consumption of red wine, apples, star fruit, or citrus fruits/juices including blood oranges (with the exception of oranges, mandarins, and lemons) within 7 days before the first dose of the investigational product.

No subject who participated in Part A of this study participated in Part B.

### Interventions

Subjects who met all of the inclusion/none of the exclusion criteria were eligible to participate in the study. All enrolled subjects were admitted to the Clinical Unit on the evening of Day 1 (evening prior to dosing).

For Part A, for each of the two treatment periods, subjects remained in the Unit until 48 h postdosing, and were discharged following the last postdose assessment, if no clinically significant abnormalities were noted. Treatment periods were separated by at least a 7‐day washout.

For Part B, all subjects were administered daprodustat on Day 1 with pharmacokinetic blood sampling over the next 48 h. Starting the morning of Day 3, all subjects were administered 200‐mg trimethoprim twice‐daily for 3 days (i.e., through Day 5). On Day 6 subjects were administered concomitantly daprodustat with 200 mg trimethoprim in the morning, with pharmacokinetic blood sampling occurring over the next 48 h. Trimethoprim continued to be administered at 12 h postdosing on Day 6, and twice daily on Day 7. Subjects were discharged from the Unit on Day 8 following the last postdose assessment, if no clinically significant abnormalities were noted.

For both parts, all investigational products were administered by Clinical Unit staff who confirmed compliance.

### Assessments and statistical analysis method

#### Pharmacokinetic

##### Part A

Blood samples for pharmacokinetic (PK) analyses were collected on Day 1 of each treatment period predose and at 0.5, 1, 1.5, 2, 3, 4, 6, 8, 10, 12, 24, 36, and 48 h postdose.

##### Part B

Blood samples for PK analyses were collected on Day 1 and Day 6 predose and at 0.25, 0.5, 1, 2, 3, 4, 5, 6, 8, 12, 18, 24, and 48 h postdose.

Plasma analysis was performed under the management of Bioanalytical Science and Toxicokinetics, Drug Metabolism and Pharmacokinetics (DMPK), and GlaxoSmithKline (GSK). Samples were collected at nominal times relative to the proposed time of pioglitazone/rosuvastatin and daprodustat dosing. Blood samples were collected into K3 EDTA tubes and immediately placed on water ice. Samples were then centrifuged at 2000*g* for 10 min; the supernatant plasma was transferred to a Nunc^®^ tube and stored at −20°C before shipment. Samples were shipped frozen to PPD (Middleton, WI) where plasma samples were analyzed for rosuvastatin, pioglitazone, or daprodustat and predominant metabolites (GSK2391220 (M2), GSK2531403 (M3), GSK2487818 (M4), GSK2506102 (M5), GSK2531398 (M6), and GSK2531401 (M13)).

For the analyses to be acceptable, no more than one third of the QC results were to deviate from the nominal concentration by more than 15%, and at least 50% of the results from each QC concentration should be within 15% of nominal. The applicable analytical runs met all predefined run acceptance criteria.

Pharmacokinetic analysis was performed using Phoenix WinNonLin^®^ 6.3 (A Certara Company, Princeton, N.J). Pharmacokinetic parameters were determined from concentration–time data for pioglitazone, rosuvastatin, daprodustat, and predominant metabolites of daprodustat using standard noncompartmental methods. The pharmacokinetic parameters of interest for each treatment were AUC_0‐∞_ (area under the concentration–time curve from time zero (predose) extrapolated to infinite time), *C*_max_ (maximum observed concentration), AUC_0‐t_ (area under the concentration–time curve from time zero (predose) to the time of the last quantifiable concentration), *t*_max_ (time of occurrence of *C*_max_), and *t*" (terminal phase half‐life), as data permitted.

Following log_e_ transformation, AUC_0‐∞_ [defaulting to AUC_0‐t_ if AUC_0‐∞_ could not be consistently determined] and *C*_max_ of pioglitazone and rosuvastatin (for Part A) or daprodustat and six predominant metabolites (for Part B) were separately analyzed using a mixed‐effects model with fixed‐effect terms for treatment and period, and subject as a random effect in the model. Point estimates and their associated 90% confidence intervals were constructed separately for the difference test reference.

As data permitted, *t*_max_ was analyzed nonparametrically for Parts A and B using the Wilcoxon's Matched Pairs Method. The point estimates and 90% confidence intervals for the median differences were calculated for the difference test reference.

No formal hypothesis was tested in this study. For each primary PK endpoint, point estimates and corresponding 90% confidence intervals were constructed for the ratio of the geometric least‐squares (LS) mean of the test treatment to the geometric LS mean of the reference treatment.

The comparisons of interest were as follows:

For Part A, 15 mg pioglitazone tablet and 10 mg rosuvastatin tablet + 100 mg daprodustat tablet (test) versus 15 mg pioglitazone tablet and 10 mg rosuvastatin tablet (reference). For Part B, two 100 mg trimethoprim tablets + 25 mg daprodustat tablet (test) versus 25 mg daprodustat tablet (reference).

### Tolerability measures

Tolerability measures included adverse events (AEs), serious adverse events (SAEs), clinical laboratory findings, vital signs, and concurrent medication assessments. An AE was defined as any untoward medical occurrence in a subject or clinical investigation subject, temporally associated with the use of an investigational product, whether or not considered related to the investigational product. An SAE was defined as any untoward medical occurrence that, at any dose, resulted in death, was life threatening, required hospitalization or prolonged existing hospitalization, resulted in disability/incapacity, was a congenital anomaly/birth defect or was associated with liver injury and impaired liver function. The investigator or site staff was responsible for detecting, documenting, and reporting events that met the definition of an AE or SAE.

## Results

### Subjects

This study was conducted between 11 February 2015 and 12 August 2015. A total of 40 healthy, adult male (25) and female (15) subjects were enrolled in this study, with 13 males and 7 females in Part A, and 12 males and 8 females in Part B. The mean age (standard deviation) in Part A was 35 (14) years, and in Part B was 32 (11) years. One subject was withdrawn from Part A, while one subject was withdrawn in Part B while all other subjects completed all treatment periods and assessments. Of the subjects that withdrew from the study, both were withdrawn due to a protocol violation: in Part A the subject took a prohibited medication prior to enrollment but within the 30 days prior to screening; in Part B the subject was withdrawn due to a positive urine drug screen (benzodiazepines). All other subjects completed all treatment periods and assessments. Demographic characteristics of the study population can be found in Table 1.

**Table 1:** Demographic data and subject disposition

Parameter		Part A *N* = 20	Part B *N* = 20
Age, years	Mean ± SD min‐max	35 ± 14 18–58	32 ± 11 18–54
Gender, *n*	Male (%) female (%)	13 (65%) 7 (35%)	12 (60%) 8 (40%)
Ethnicity, *n*	Hispanic or Latino (%) not Hispanic or Latino (%)	2 (10%) 18 (90%)	0 20 (100%)
Height, cm	Mean ± SD min‐max	173 ± 11 156–191	172 ± 11 156–188
Weight, kg	Mean ± SD min‐max	78.4 ± 15.1 53.3–104.7	76.7 ± 13.4 54.6–99.1
BMI, kg/m^2^	Mean ± SD min‐max	26.1 ± 2.7 19.9–29.5	25.8 ± 3.1 19.5–30.0
Completion status, *n*	Completed (%) Withdrawn (%)	19 (95%) 1 (5%)	19 (95%) 1 (5%)

### Pharmacokinetics

#### Daprodustat as a perpetrator

##### Pioglitazone

**Figure 1:**
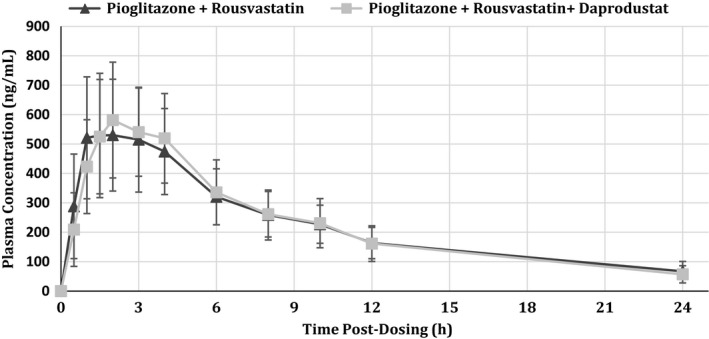
Mean (±SD) plasma concentrations of pioglitazone following administration of 15 mg pioglitazone with 10 mg rosuvastatin (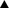) or 15 mg pioglitazone with 10 mg rosuvastatin and 100 mg daprodustat (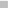).

Peak concentrations of pioglitazone were rapidly achieved, both with and without concomitant administration of 100‐mg daprodustat, with observed maximum plasma concentrations 2 h for both treatment periods (Fig. 1). Median elimination half‐life values were similar (7.93 h for pioglitazone and daprodustat and 7.01 h for pioglitazone alone) for the two treatment periods (data not shown). The geometric LS mean *C*_max_ for pioglitazone achieved in this study was 616.6 ng/mL for the test treatment, while for the reference treatment it was 546.5 ng/mL (Table 2). The geometric LS mean ratio (90% confidence interval) for the test:reference comparison for AUC_0‐∞_ was 0.98 (0.91, 1.04) while for *C*_max_ it was 1.13 (1.01, 1.25) (Table 2).

**Table 2:** Summary of statistical analysis results of pioglitazone (top) and rosuvastatin (bottom) pharmacokinetic parameters

Pioglitazone pharmacokinetics			
Parameter	Test	Reference	Geometric LS mean ratio (90% CI)
AUC_0‐t_ng.h/mL	5095.00 (31.1)	5064.85 (28.8)	1.00 (0.93, 1.07)
AUC_0‐∞_ng.h/mL	5758.58 (28.5)	5911.88 (33.1)	0.98 (0.91, 1.04)
*C*_max_ng/mL	616.6 (29.8)	546.5 (35.1)	1.13 (1.01, 1.25)

Rosuvastatin pharmacokinetics			
Parameter	Test	Reference	Geometric LS Mean Ratio (90% CI)
AUC_0‐t_ng.h/mL	40.30 (43.6)	43.20 (44.2)	0.96 (0.86, 1.08)
AUC_0‐∞_ng.h/mL	48.46 (42.5)	48.52 (39.7)	1.03 (0.91, 1.17)
*C*_max_ng/mL	4.68 (42.2)	5.04 (45.7)	0.94 (0.83, 1.07)

Parameters are presented as geometric least‐squares mean (%CV – within‐subject coefficient of variation). Test treatment was 15 mg pioglitazone tablet and 10 mg rosuvastatin tablet + 100 mg daprodustat tablet while reference treatment was 15 mg pioglitazone tablet and 10 mg rosuvastatin tablet. LS, Least squares.

##### Rosuvastatin

**Figure 2:**
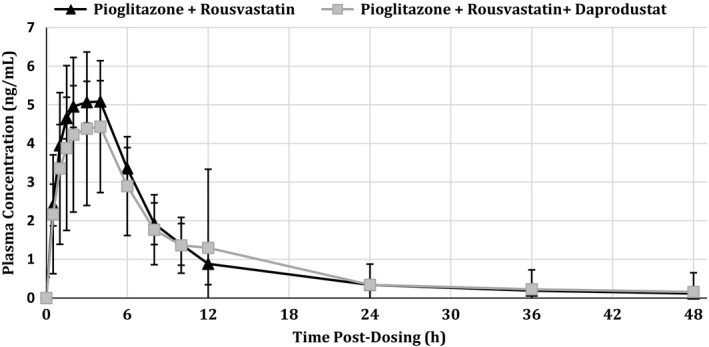
Mean (±SD) plasma concentrations of rosuvastatin following administration of 15 mg pioglitazone with 10 mg rosuvastatin (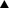) or 15 mg pioglitazone with 10 mg rosuvastatin and 100 mg daprodustat (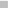).

Peak plasma concentrations of rosuvastatin were also rapidly achieved, both with and without concomitant administration of 100 mg daprodustat, with maximum observed plasma concentrations 4 h for both treatment periods (Fig. 2). Median elimination half‐life values were generally similar (21.3 h for rosuvastatin and daprodustat and 15.6 h for rosuvastatin alone) for the two treatment periods (data not shown). The geometric LS mean *C*_max_ for rosuvastatin achieved in this study was 4.68 ng/mL for the test treatment, while for the reference treatment it was 5.04 ng/mL (Table 2). The geometric LS mean ratio (90% confidence interval) for the test:reference comparison for AUC_0‐∞_ was 1.03 (0.91, 1.17) while for *C*_max_ it was 0.94 (0.83, 1.07) (Table 2).

#### Daprodustat as a victim

**Figure 3:**
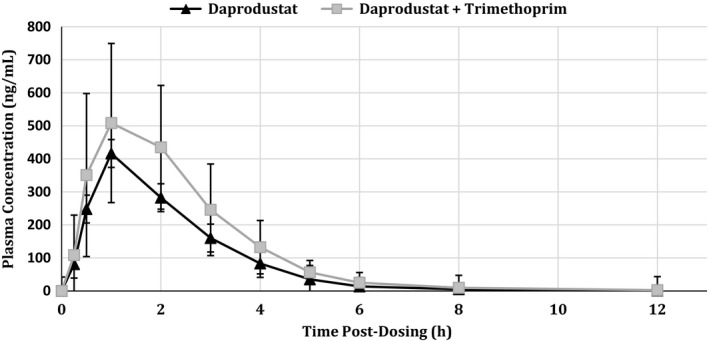
Mean (±SD) plasma concentrations of rosuvastatin following administration of 15 mg pioglitazone with 10 mg rosuvastatin (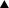) or 15 mg pioglitazone with 10 mg rosuvastatin and 100 mg daprodustat (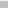).

Plasma concentrations of daprodustat achieved observed peak levels, both with and without concomitant administration of steady‐state trimethoprim, at 1 h for both treatment periods (Fig. 3). Median elimination half‐life values were 4.48 h for daprodustat and trimethoprim and 3.06 h for daprodustat alone (data not shown). The geometric LS mean *C*_max_ for daprodustat was 559.7 ng/mL for the test treatment, while for the reference it was 441.8 ng/mL (Table 3). The geometric LS mean ratio (90% confidence interval) for the test:reference comparison for AUC_0‐∞_ was 1.48 (1.39, 1.59) while for *C*_max_ it was 1.28 (1.09, 1.51) (Table 3). Of the metabolites measured, GSK2531401 (M13) showed the greatest deviation from unity in the geometric LS mean ratio (Table 4). The ratio for AUC_0‐∞_ was 0.68 (0.63, 0.73) while for *C*_max_ it was 0.60 (0.54, 0.67); all other ratios for AUC_0‐∞_ were between 1.05 (GSK2487818; M4) and 0.96 (GSK2506102; M5) while for *C*_max_ the ratios were between 0.93 (GSK2487818) and 0.85 (GSK2506102).

**Table 3:** Summary of statistical analysis results of daprodustat pharmacokinetic parameters

Parameter	Test	Reference	Geometric LS mean ratio (90% CI)
AUC_0‐t_ng.h/mL	1371.81 (36.8)	935.96 (36.1)	1.48 (1.39, 1.59)
AUC_0‐∞_ng.h/mL	1373.70 (36.9)	936.86 (36.1)	1.48 (1.39, 1.59)
*C*_max_ng/mL	559.7 (31.3)	441.8 (46.7)	1.28 (1.09, 1.51)

Parameters are presented as geometric least‐squares mean (%CV – within‐subject coefficient of variation). Test treatment was two 100 mg trimethoprim tablets + 25 mg daprodustat tablet while reference treatment was 25 mg daprodustat tablet. LS, Least squares.

**Table 4:** Summary of statistical analysis results of daprodustat metabolite pharmacokinetic parameters

Metabolite	Parameter	Test	Reference	Geometric LS mean ratio (90% CI)
GSK2391220 (M2)	AUC_0‐*t*_ ng.h/mL	157.33 (30.74)	160.95 (32.5)	0.99 (0.95, 1.04)
	AUC_0‐∞_ng.h/mL	158.44 (30.3)	161.82 (32.5)	0.99 (0.95, 1.04)
	*C*_max_ng/mL	31.27 (27.4)	35.21 (33.4)	0.90 (0.85, 0.96)
GSK2531403 (M3)	AUC_0‐*t*_ng.h/mL	148.03 (26.9)	152.68 (29.5)	0.98 (0.94, 1.03)
	AUC_0‐∞_ng.h/mL	149.09 (26.8)	153.55 (29.5)	0.98 (0.94, 1.03)
	*C*_max_ ng/mL	28.48 (24.6)	32.70 (30.7)	0.89 (0.83, 0.94)
GSK2487818 (M4)	AUC_0‐*t*_ng.h/mL	115.37 (32.0)	110.83 (33.3)	1.05 (1.01, 1.09)
	AUC_0‐∞_ng.h/mL	116.08 (31.8)	111.53 (33.2)	1.05 (1.01, 1.09)
	*C*_max_ng/mL	27.44 (25.9)	29.97 (33.6)	0.93 (0.86, 1.00)
GSK2506102 (M5)	AUC_0‐*t*_ng.h/mL	34.86 (22.9)	36.73 (26.1)	0.96 (0.91, 1.01)
	AUC_0‐∞_ng.h/mL	35.49 (22.8)	37.54 (26.0)	0.96 (0.91, 1.00)
	*C*_max_ng/mL	6.53 (22.5)	7.77 (27.1)	0.85 (0.80, 0.91)
GSK2531398 (M6)	AUC_0‐*t*_ng.h/mL	76.85 (24.8)	75.53 (28.8)	1.03 (0.98, 1.07)
	AUC_0‐∞_ng.h/mL	77.59 (24.7)	76.34 (28.4)	1.02 (0.98, 1.07)
	*C*_max_ng/mL	15.09 (22.3)	17.10 (29.7)	0.89 (0.84, 0.95)
GSK2531401 (M13)	AUC_0‐*t*_ng.h/mL	83.63 (30.1)	126.06 (28.1)	0.68 (0.62, 0.73)
	AUC_0‐∞_ng.h/mL	84.53 (30.1)	126.84 (28.0)	0.68 (0.63, 0.73)
	*C*_max_ng/mL	13.89 (34.1)	23.46 (32.6)	0.60 (0.54, 0.67)

Parameters are presented as geometric least‐squares mean (%CV – within‐subject coefficient of variation). Test treatment was two 100 mg trimethoprim tablets + 25 mg daprodustat tablet while reference treatment was 25 mg daprodustat tablet. LS, Least Squares.

### Tolerability

There were no SAEs or deaths reported during the study, including up to 14 days following the last dose of study treatment, and no withdrawals due to adverse events (AEs). A total of 3 subjects (15%) reported an AE during Part A of the study, while 9 subjects (45%) reported an AE in Part B (Table 5).

**Table 5:** Summary of all adverse events

Part A	Regimen A (*n* = 20)	Regimen B (*n* = 19)
Preferred Term	*n* (%)	*n* (%)
Upper respiratory tract infection	0	2 (11)
Headache	1 (5)	1 (5)

Part B	Regimen C (*n* = 20)	Regimen D (*n* = 19)
Preferred Term	*n* (%)	*n* (%)
Diarrhea	2 (10)	1 (5)
Constipation	1 (5)	1 (5)
Nausea	0	2 (11)
Aphthous stomatitis	0	1 (5)
Tongue coated	0	1 (5)
Vomiting	1 (5)	0
Vulvovaginal mycotic infection	0	2 (11)
Rhinitis	0	1 (5)
Catheter site phlebitis	0	1 (5)

Descriptions of regimens: A = 15 mg pioglitazone + 10 mg rosuvastatin; B = 15 mg pioglitazone + 10 mg rosuvastatin + 100 mg daprodustat; C = 25 mg daprodustat; D = 25 mg daprodustat + 200 mg trimethoprim BID for 5 days.

For Part A, a slightly higher proportion of subjects administered pioglitazone, rosuvastatin, and 100‐mg daprodustat concomitantly reported AEs (11%) as compared to subjects administered pioglitazone and rosuvastatin concomitantly (5%). The most commonly reported AE was upper respiratory tract infection, reported by two (11%) subjects administered pioglitazone, rosuvastatin, and 100‐mg daprodustat concomitantly. All other AEs were single‐subject reports.

For Part B, a slightly higher proportion of subjects administered trimethoprim and 25‐mg daprodustat concomitantly reported AEs (47%) as compared to subjects administered daprodustat alone (10%). The most commonly reported AEs were both reported following administration of trimethoprim and 25‐mg daprodustat concomitantly: Nausea, reported by 2 (11%) subjects, and vulvovaginal mycotic infection, reported by 2 (11%) subjects. Diarrhea was the next most commonly reported AE, experienced by 2 (10%) subjects administered 25‐mg daprodustat alone. All other AEs were single‐subject reports.

## Discussion

The purpose of this study was to assess the potential for daprodustat, which has both CYP2C8 and OATP1B1 inhibitory activity in vitro, to affect the pharmacokinetics of pioglitazone, a CYP2C8 probe substrate, and rosuvastatin, an OATP1B1 probe substrate. In addition, the effect of trimethoprim, a weak CYP2C8 inhibitor, on the pharmacokinetics of daprodustat was also assessed. The results of this study were twofold: First, 100‐mg daprodustat, when coadministered with either pioglitazone or rosuvastatin, did not affect the pharmacokinetics of either probe substrate, as evidenced by the 90% confidence interval for the geometric LS mean ratios for both *C*_max_ and AUC being within an 0.80–1.25 no effect boundary; Second, trimethoprim coadministration led to a 48% increase in AUC with a 28% increase in *C*_max_.

In this study both pioglitazone and rosuvastatin were administered as a ‘probe cocktail’ to assess the potential of daprodustat to affect the pharmacokinetics of drugs that are either CYP2C8 or OATP1B1/BCRP substrates. It has been previously demonstrated that the pharmacokinetics of pioglitazone and rosuvastatin were not affected after coadministration as part of a 7‐probe cocktail (Rizwan et al. 2010). The results of that study support the coadministration of pioglitazone and rosuvastatin as a probe cocktail to assess the inhibitory potential of agents on compounds metabolized by the CYP2C8 or OATP1B1/BCRP pathways.

The daprodustat dose used in this study (100 mg) is approximately fourfold higher than the highest once daily therapeutic dose currently under investigation in Phase 3 studies in the treatment of patients with anemia associated with chronic kidney disease (24 mg). Even at this high dose, the average plasma *C*_max_ value following single‐dose administration is approximately 4.5 *μ*mol/L which is less than the in vitro‐derived IC_50_ values for inhibition of CYP2C8 (21 *μ*mol/L) and OATP1B1 (6 *μ*mol/L) by daprodustat.

Both CYP2C8 and CYP3A4 are the primary enzymes which catalyze the oxidative metabolism of pioglitazone (Eckland and Danhof 2000). Administration of pioglitazone with gemfibrozil, a strong inhibitor of CYP2C8, led to a marked increase (3.4‐fold) in AUC_0‐∞_, supporting a key role for CYP2C8 in the metabolism of pioglitazone (Deng et al. 2005). Although repaglinide is recognized as a more sensitive probe substrate for CYP2C8, pioglitazone was used in this study as there is a decreased potential to cause hypoglycemia. The apparent lack of effect of daprodustat, at a dose of 100 mg, suggests that there is a very low potential for a drug interaction between daprodustat and drugs that are metabolized by CYP2C8, and that they can be safely coadministered.

Metabolism is reported to be a minor route of clearance for rosuvastatin, with OATP1B1 and BCRP affecting transport across the hepatocyte membrane (Martin et al. 2003; US Department of Health and Human Services Food and Drug Administration Center for Drug Evaluation and Research (CDER), 2016). Similar to the results observed with pioglitazone, there did not appear to be an effect of daprodustat coadministration on the pharmacokinetics of rosuvastatin, suggesting that daprodustat at doses up to 100 mg can be coadministered with substrates of both OATP1B1 and/or BCRP.

Daprodustat exposure has been shown to increase markedly (4‐fold increase in *C*_max_ and a 19‐fold increase in AUC) with coadministration of gemfibrozil, a strong CYP2C8 inhibitor (Johnson et al. 2013). Furthermore, *C*_max_ and AUC of the primary metabolites of daprodustat were reduced by at least 90% and 67%, respectively. Trimethoprim has been characterized as a weak CYP2C8 inhibitor, increasing AUC ≥ 1.25‐ but < 2‐fold (US Department of Health and Human Services Food and Drug Administration Center for Drug Evaluation and Research (CDER), 2016). Coadministration of trimethoprim with rosiglitazone, a sensitive CYP2C8 probe increased rosiglitazone AUC by 1.31‐fold, consistent with trimethoprim classification as a weak inhibitor (Hruska et al. 2005). In this study, coadministration of trimethoprim with daprodustat led to a 1.28‐fold increase in *C*_max_ and 1.48‐fold increase in AUC, which is also consistent with weak CYP2C8 inhibition by trimethoprim. The effect observed on the primary metabolites of daprodustat was also minor. AUC and *C*_max_ were decreased by 32% and 40%, respectively, for metabolite GSK 2531401 while only slight decreases in *C*_max_ of approximately 7–15% were observed for the other metabolites with coadministration of trimethoprim. Based on a dose–response evaluation of daprodustat in patients with anemia associated with chronic kidney disease and the necessity to titrate daprodustat to achieve an appropriate hemoglobin target, these increases in exposure are not considered to be clinically significant (Johnson et al. 2013; Cobitz et al. 2016; Holdstock et al. 2016b).

The safety and tolerability profile following coadministration of daprodustat with either pioglitazone/rosuvastatin or steady‐state trimethoprim did not differ substantially from administration of either probes alone. All of the adverse events that were reported by the study participants were either single events or reported by no more than two subjects. Therefore, there did not appear to be any additional safety concerns from administration of daprodustat with either pioglitazone/rosuvastatin or trimethoprim.

## Conclusions

1. Coadministration of 100‐mg daprodustat with 15‐mg pioglitazone and 10‐mg rosuvastatin had no effect on pioglitazone or rosuvastatin pharmacokinetics, suggesting that drugs metabolized by CYP2C8 and OATP1B1/BCRP substrates can be coadministered with up to 100 mg of daprodustat.
2. Coadministration of daprodustat with steady‐state trimethoprim led to nonclinically significant increases in the exposure of daprodustat suggesting that daprodustat can be coadministered with weak CYP2C8 inhibitors with no dosage adjustment.
3. Administration of daprodustat concomitantly with pioglitazone and rosuvastatin, or with trimethoprim was generally well tolerated and was consistent with the safety profile seen in other studies in this population.

## Disclosures

All authors were employees of GlaxoSmithKline at the time of conduct of this study. This study was supported by GlaxoSmithKline.
